# Divergent response of grassland aboveground net primary productivity and precipitation utilization efficiency to altered precipitation patterns by process-based model

**DOI:** 10.3389/fpls.2025.1487907

**Published:** 2025-01-30

**Authors:** Chen Cheng, Lu Wu, Hongyan Liu, Boyi Liang, Xinrong Zhu, Feiyun Yang

**Affiliations:** ^1^ College of Ecology, Lishui University, Lishui, China; ^2^ China Meteorological Administration Training Center, Beijing, China; ^3^ College of Urban and Environmental Science, Peking University, Beijing, China; ^4^ College of Forestry, Beijing Forestry University, Beijing, China

**Keywords:** aboveground net primary production, APSIM model, grassland, Inner Mongolia Autonomous Region, precipitation, precipitation utilization efficiency

## Abstract

The functioning of ecosystem services in water-limited grassland ecosystems is significantly influenced by precipitation characteristics. This study aims to quantitatively assess the impact of different precipitation scenarios on grassland productivity using the APSIM model. Historical weather data from 1968 to 2017 and observational data from three types of steppes (meadow, typical, and desert steppe) in Inner Mongolia Autonomous Region from 2004 to 2010 were collected to determine key crop variety parameters for the APSIM model. The effects of annual precipitation, seasonal precipitation, and inter-growing season precipitation variability on aboveground net primary production (ANPP) and precipitation utilization efficiency (PUE) in different types of steppes were investigated by scenario simulation by validated model. The simulated ANPP shows distinctive responses to the changed rainfall characteristics, where the influence of precipitation decreasing is more evident than precipitation increasing by the same precipitation change. Regarding steppe types, the typical steppe responded more strongly to increased precipitation, while decreased precipitation led to higher decline in ANPP for desert steppe. Precipitation during growing seasons caused more significant change than dormancy seasons regarding ANPP, however, PUE show the opposite trend, indicating the contribution of unit level precipitation changes to productivity is significant during dormancy seasons. The effect of changing precipitation during middle growing season outweighed that of late growing season and early growing season, and the positive effect of increasing precipitation were more pronounced in typical steppe and desert steppe if facing early growing season precipitation increase in the future. The research results provide a theoretical basis and technical support for optimizing grassland production management.

## 1 Introduction

Grassland, as an important component of terrestrial ecosystems, accounts for approximately 70% of the world’s available agricultural production area (Mo et al., 2012). China has a natural usable grassland area of 331 million hectares, of which alpine meadows have the largest area, about 59 million hectares, accounting for 17% of the total area (Mo et al., 2012). Next are temperate steppe, alpine steppe, and temperate desert steppe, all accounting for about 10% of the total grassland area in China (Tian et al., 2020). Semi-arid steppe is a major type of terrestrial ecosystem worldwide, with an area of approximately 9.1×10^6^ km^2^ (Liu M. et al., 2017). Except for an increase in the area of typical steppe, the area of other grassland types has decreased (Zhu et al., 2020; Tian et al., 2020). Currently, the functioning of grassland ecosystems is influenced by climate change, which can lead to changes in biological habitats and even a reduction in global biodiversity (Qie et al., 2019). The ecological service functions of grassland ecosystems are particularly affected by precipitation characteristics in water-limited grassland ecosystems, which is more pronounced (Petrie et al., 2018; Zhang et al., 2018; Ru et al., 2022). It is urgent to assess the impact of different precipitation scenarios on grassland productivity.

Precipitation characteristics, such as precipitation pulse, precipitation intensity, precipitation duration, and precipitation frequency, have important effects on grassland productivity (Guan et al., 2018). Guo et al. (2015) explored the impact of different precipitation scenarios on gross primary productivity (GPP) of alpine meadows using eddy covariance technology, and found that precipitation intensity and precipitation duration are the main meteorological factors affecting GPP. Hoover and Rogers (2016) studied the impact of interannual precipitation on ecosystem carbon storage and cycling, and found that short-term episodic droughts are more likely to cause carbon loss than long-term continuous droughts. Andrew et al. (2020) found that increasing precipitation during the dry growing season reduces precipitation utilization efficiency (PUE) and aboveground net primary productivity (ANPP) by eliminating the temporal variation of extreme climate season precipitation. However, few studies have focused on the impact of precipitation on grassland productivity for intra-seasons, e.g. during the mid-late growing season, which plays a crucial role in controlling carbon and nitrogen cycling and biological interactions (Li P. et al., 2018; Yuan and Yang, 2021).

As precipitation can affect soil water content and subsequently influence vegetation phenology and growth cycles, extreme precipitation in arid grassland areas has a greater impact on grassland productivity than extreme temperature (Iturrate-Garcia et al., 2016). Although increased precipitation can alleviate the pressure of terrestrial water storage, promote water infiltration, and prevent premature wilting of grassland vegetation (Liu M. et al., 2017; Hao et al., 2017), they may also increase grassland ecosystem respiration consumption and surface runoff (Guo et al., 2015; Felton et al., 2018). The uncertainty of grassland productivity under different precipitation scenarios of different types of steppes still needs to be further investigated.

Previous studies have explored the impact of precipitation on grassland productivity through limited years of field experiments (<3 years), and whether there are legacy effects of precipitation characteristics requires verification on longer time scales (Bodner and Robles, 2017; Wang et al., 2018). Crop models can be used to simulate the impact of different precipitation scenarios on grassland productivity and solve scientific issues such as wastage of funding resources, lengthy duration, and timeliness of technological applications in actual precipitation scenario experiments. Currently, most existed vegetation models have been used to simulate the dynamic growth of different plant functional types, and their response to climate change (Susanne et al., 2018). Severe soil degradation in Inner Mongolia Autonomous Region, especially the degradation of 0-20cm layers constrained vegetation growth, while in the typical vegetation model, e.g. DGVMs (Krause et al., 2018), soil was divided into two layers (0-0.5m for top layer and 0.5-1.0m for deeper layer), which may neglect the effect of top soil layers. The APSIM model has been widely used to address the limitations of traditional field experiments, such as spatial and temporal constraints, limited research subjects, and long experimental cycles with more detailed soil layer definition may solve this problem (Holzworth et al., 2014; Gao et al., 2022; Liu et al., 2022; Gong et al., 2023; Wu et al., 2024). Existed studies demonstrate using APSIM model to simulate the impact of different precipitation scenarios on crop yields is effective, such as wheat (Gao et al., 2022), corn (Gong et al., 2023), and potatoes (Liu et al., 2022), which provide theoretical guidance and technical support for optimizing crop water and fertilizer management measures at the regional scale. Therefore, we can utilize the advantages of the APSIM model to evaluate the response of grassland ecosystems to precipitation changes.

In this study, historical meteorological data from 1968 to 2017 and observational data of three types of steppes (meadow, typical, and desert steppe) from 2004 to 2010 in Inner Mongolia Autonomous Region were collected to determine the key crop variety parameters of the APSIM model. The effects of annual, seasonal, and intra-growing season precipitation changes on ANPP and PUE of different types of steppes were explored through scenario simulation based on validated model. The results of this study will provide a theoretical basis and technical support for optimizing grassland production management.

## 2 Materials and methods

### 2.1 Study area

The present study was conducted at three types of grassland sites in Inner Mongolia Autonomous Region, namely E’erguna Qi (50.47°N, 120.41°E, meadow steppe (MS) dominated by Leymus chinensis and *Stipa baicalensis*), Xilinhot (43.63°N, 116.70°E, typical steppe (TS) dominated by *Leymus chinensis*), and Siziwang Qi (42.16°N, 111.60°E, desert steppe (DS) dominated by *T. angustifolia*, *Artemisia scoparia*, and *Cleistogenes squarrosa*) (Piao et al., 2007; Li QY. et al., 2018; Hou et al., 2023). The MS is located in the northeastern part of Inner Mongolia, with an average annual temperature of -2.07 ± 1.08°C and an average annual precipitation of 358.7 ± 85.7 mm. The TS is situated in the central region of Inner Mongolia, with an average annual temperature of 3.19 ± 0.99°C and an average annual precipitation of 281.5 ± 88.6 mm. The DS is found in the western part of Inner Mongolia, with an average annual temperature of 4.25 ± 0.98°C and an average annual precipitation of 316.4 ± 77.3 mm. The annual NPP was highest for meadow steppe, which was ~250 g C/m^2^, followed by typical steppe (~150 g C/m^2^), and the lowest in desert steppe (<100 g C/m^2^) (Hossain et al., 2021). The community height followed the same patter as annual NPP in the three steppes, with community height>23 cm in meadow steppe while community height at <14cm for desert steppe in August (Zhang and Ren, 2023). The mean LAI values in meadow steppe were 1.45 m^2^/m^2^, while in typical steppe, the LAI values had a distribution range of 0.34~2.34 m^2^/m^2^ with a mean value of 1.18 m^2^/m^2^, and for that of desert steppe, mean LAI was 0.57 m^2^/m^2^ (Shen et al., 2022). The starting of growing season (SOS) in desert steppe was around early April while in typical steppe, plant started growth by middle April. For meadow steppe, SOS occurred in early May. The end of growing season was 260~270 (DOY) in meadow steppe, 270~280 in typical steppe and 280~300 in desert steppe (Wang et al., 2019).

### 2.2 Data sources

The data for this study mainly includes meteorological, soil, and vegetation growth management data. Meteorological data comes from the official website of China Meteorological Administration (https://data.cma.cn/). The meteorological station data near the selected types of steppe sites, including MS, TS, and DS, with station codes 50425, 54102, and 53362 respectively, provides key indicators such as daily maximum temperature, daily minimum temperature, daily precipitation, sunshine duration, wind speed, relative humidity, solar radiation, and sun hours data for the period from 1968 to 2017. The calculation of sunshine duration and solar radiation indicators were based on the method proposed by Wu et al. (2017). The data of the top 0-20 cm soil layer is measured on-site (**Table 1**), while the data of the deep 20-100 cm soil layer is derived from the global soil data grid product (Wang et al., 2017). The normalized soil physicochemical properties data specific to steppe sites were extracted, including soil texture (sand, silt, and clay content), bulk density, soil organic matter, and soil pH. The natural grassland sites were fenced in the early 1980s with an area of 5 km × 5 km (Wu et al., 2022). Plant growth data includes green-up date, flowering date, as well as aboveground net primary production (ANPP, measured as above-ground biomass which was cut at 5 cm) from May to September with 1 m × 1 m plots were obtained from the animal husbandry experimental stations of China Meteorological Administration.

**Table 1:** Physical and chemical characteristics of 0-20cm surface soil at three representative grassland stations.

Grassland type	Experiment station	Sand/%	Silt/%	Clay/%	ASW/DUL-Wilting	Soil bulk density/g cm^-3^	pH
Meadow steppe	E’erguna Qi	44.5	41.7	13.7	44mm	1.16	8.30
Typical steppe	Xilinhot	40.4	41.8	17.8	43mm	1.34	7.90
Desert steppe	Siziwang Qi	76.8	14.2	12	14.6mm	1.44	7.87

### 2.3 Parameter determination and validation results of APSIM model

APSIM (Agricultural Production Systems Simulator) is a modular modeling framework that reconfigures individual modules for crop production and soil management to simulate aboveground net primary productivity (Liu et al., 2022; Gong et al., 2023). The plant simulation module of APSIM is versatile (Wu et al., 2024), and an independent crop growth, soil water, and soil nitrogen module are built using a ‘plug-and-play’ structure. Based on the lucerne module, perennial module parameters and crop parameters are determined, and a natural grassland community plant module is developed. This study utilizes the APSIM-SoilN module for soil carbon and nitrogen, the APSIM-SoilWat module for soil water, the APSIM-Surface Organic Matter module for crop residue, and the crop module (Probert et al., 1998; Wang et al., 2002; Holzworth et al., 2014). Previous studies have validated the accuracy of APSIM in simulating ANPP in meadow steppe and typical steppe (Wu et al., 2022), demonstrating well acceptable simulation accuracy. In this study, an optimization method is further applied to calibrate the genetic parameters of desert steppe using measured ANPP data from 2004-2010, and the response of the three types of steppes to changes in precipitation is assessed based on the validated APSIM model.

Based on the initial soil parameters (**Table 1**) and experimental data from 2004-2010, three types of community vegetation genetic parameters for the three steppes were determined through trial-and-error method, while the model variety parameters for meadow steppe and typical steppe were inherited from previous studies (Wu et al., 2022). The new calibrated model variety parameters for desert steppe shown in **Table 2**.

**Table 2:** Key parameters of desert steppe community.

Model parameter	Units	Meaning	Default_value	Desert steppe
T_base_	°C	Base temperature for calculating daily effective thermal time below which the temperature is regarded as non-effective.	1	3 (Chen et al., 2014)
x_pp_end_of_juv	h	Change of day length between the end of juvenile stage to floral initiation	12.3-14.0	12.3-14.0
y_tt_end_of_juv	°C · d	Thermal time required from end of juvenile stage to floral initiation stage related to day length change	260-160	160-160
f_sw	—	Fraction of available soil water maximum soil water in the profile, 0.0 indicates no water available, 1.0 indicates maximum water storage.	0.0-1.0	0.0-0.5
tt_stress_emerg	—	Delay in green-up date by reduction in daily thermal time calculation related to f_sw fraction change, 1.0 indcates no delay in green-up stage.	1.0-1.0	0.3-1.0
tt_emer_juven	°C · d	Thermal time accumulation required from green-up to the end of juvenile stage	700	500
stage_stem_reduction	—	Reduction in plant phenology due to mowing or harvesting	4	2
ratio_root_shoot	—	Daily photosynthate allocation between root and shoot, growth cycles from seeding duration: transition duration: reproductive duration	1.0: 0.40: 1.0	3.0: 1.0: 0.3
frac_leaf	—	Daily photosynthate of shoot allocated for leaf development, growth cycles from seeding duration: transition duration: reproductive duration	0.45	0.65
x_lai	—	Leaf area index	0.0-3.0	0.0-3.0
y_sla_max	mm^2^ g^-1^	Maximum specific leaf area for daily increase in LAI, between which the value was linear interpolated	60000-30000	25000-15000
RUE	g MJ^-1^	Radiation use efficiency, growth cycles from seeding duration: transition duration: reproductive duration	1.8:1.4: 0.8	1.15: 0.9: 0.05
Kc	pa	Transpiration efficiency coefficient change, growth cycles from seeding duration: transition duration: reproductive duration	0.006: 0.005: 0.003	0.006: 0.003: 0.001

The process for determining the model parameters is as follows: 1) Prepare model input data, including meteorological data, soil physical characteristics, and soil chemical characteristics (soil organic carbon, SOC, carbon-nitrogen ratio, CN ratio). Use soil water dynamics calculation software to calculate field capacity (mm/mm) and saturated water content (mm/mm) (Saxton and Rawls, 2006), with a default soil reflectance of 0.13. 2) Run the model in advance for 2 years to reach a stable equilibrium of soil compartments. Calibrate model parameters for the green-up stage (development stage (DVS)=3) and flowering stage (DVS=6) based on accumulated temperature during the growth and development period (Holzworth et al., 2014). Considering the impact of spring drought on the green-up stage, the temperature accumulation is adjusted from no effect (value of 1.0) to a 50% slowdown (value of 0.5). 3) Calibrate model parameters such as root/shoot ratio (RS), leaf distribution coefficient (DL), leaf area (LA), specific leaf area (SLA), radiation use efficiency (RUE), and transpiration efficiency coefficient (Kc) to account for the process of dry matter accumulation and community differences for varied vegetation (Wu et al., 2022). 4) Evaluate the performance of the APSIM model using statistical indicators such as regression coefficients (α), regression constant (β), coefficient of determination (R^2^) (Cheng et al., 2023, 2024).

**Figure 1:**
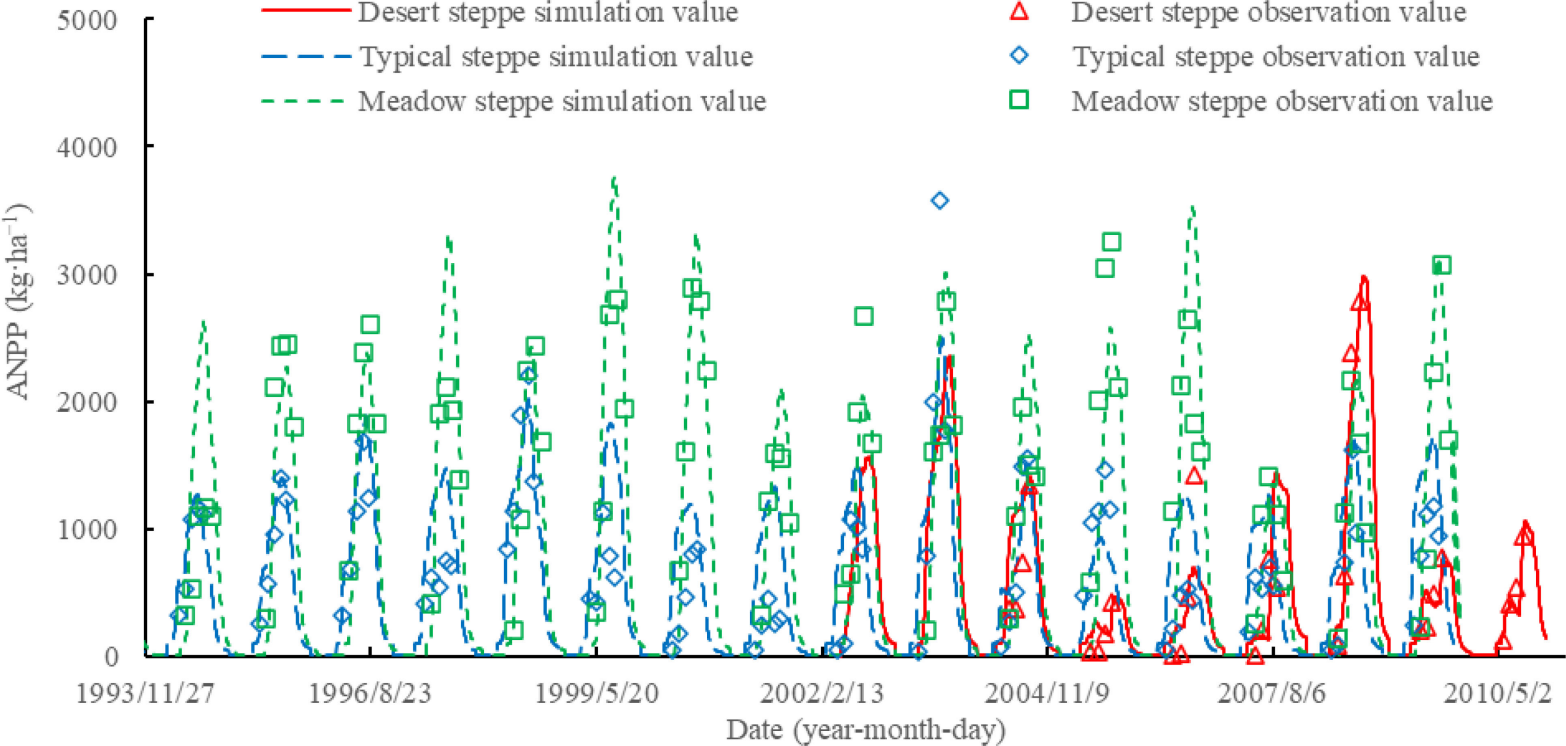
Comparison and validation of observed and simulated net primary productivity on different steppes based on APSIM model.

Based on the independent field observation data from 2004 to 2010, statistical validation of the APSIM model for grassland above-ground net primary productivity (ANPP) was obtained. The simulated ANPP for three types of steppe communities were found to be consistent with the observed values, with ${\text{X}}_{\text{sim}}=0.83*{\text{X}}_{\text{obs}}-37.86$ > (R^2^ = 0.67) (**Figure 1**). This indicates that the APSIM model can quantitatively reflect the trend of ANPP variations in the three types of steppes, thereby enabling simulated analysis of different precipitation scenarios.

### 2.4 Scenario design

Based on historical meteorological data from 1968 to 2017, we designed three different precipitation change scenarios (annual precipitation (AP), seasonal precipitation (SP), and growing season precipitation (GSP)) to explore the aboveground net primary productivity (ANPP) response to varying precipitation amounts for different types of steppes (Gao et al., 2013). The baseline indicated the precipitation of current meteorological data, three kinds of precipitation characteristics were selected for precipitation scenarios (**Table 3**). We established six levels of precipitation change gradient for the AP and twelve levels for the SP. The GSP accounts for 85% to 90% of the AP, while dormancy precipitation (DP) accounts for 10% to 15% of the AP. Furthermore, the GSP has been classified into eighteen levels of precipitation change gradient, including early, middle and late growing season precipitation (EGSP, MSSP and LGSP). For instance, AP-10% represents a daily reduction of 10% in precipitation throughout the year, whereas GSP-10% indicates a 10% reduction in daily precipitation from April to September, while there is no change in daily precipitation from October to March.

**Table 3:** Design of precipitation scenarios for three types of steppes.

Precipitation scenarios		Horizontal gradient					
		-30%	-20%	-10%	+10%	+20%	+30%
Annual precipitation (AP)		P-30%	P-20%	P-10%	P+10%	P+20%	P+30%
Seasonal precipitation (SP)	Growth season precipitation (GSP) (April to September)	G-30%	G-20%	G-10%	G+10%	G+20%	G+30%
	Dormancy precipitation (DP) (October to March)	D-30%	D-20%	D-10%	D+10%	D+20%	D+30%
Growth season precipitation (GSP)	Early growing season precipitation (EGSP) (April to May)	E-30%	E-20%	E-10%	E+10%	E+20%	E+30%
	Middle growing season precipitation (MSSP) (June to July)	M-30%	M-20%	M-10%	M+10%	M+20%	M+30%
	Late growing season precipitation (LGSP) (August to September)	L-30%	L-20%	L-10%	L+10%	L+20%	L+30%

Based on the precipitation scenarios, the change rate of net primary productivity above ground (CR_ANPP_, Equation 1), the precipitation utilization efficiency (PUE, Equation 2) during different precipitation scenarios were determined through simulation and comparison. Data analysis for significance was conducted using SPSS26. If the data followed a normal distribution, Duncan’s method was used to compare the significance differences among different treatments. If the data did not follow a normal distribution, non-parametric analysis (Kruskal-Wallis) was employed to analyze the significance of different factors.


(1) $${\text{CR}}_{\text{ANPP}}=\frac{{\text{ANPP}}_{treat}-{\text{ANPP}}_{CK}}{{\text{ANPP}}_{CK}}*100\%$$

(2) $$\text{PUE}=\frac{\left|{\text{ANPP}}_{treat}-{\text{ANPP}}_{CK}\right|}{{\text{P}}_{change}}$$

Where ${\text{ANPP}}_{treat}$ represents the maximum net primary productivity (kg·ha^−1^) of the steppe in a changed simulation scenario, using the maximum biomass of the community as a substitute. ${\text{ANPP}}_{CK}$ represents the maximum net primary productivity (kg·ha^−1^) of the steppe under the actual precipitation condition. ${\text{P}}_{change}$ refers to the absolute change of precipitation amount (mm) under varying precipitation scenarios.

## 3 Results

### 3.1 Response of different types of grassland productivity to annual precipitation changes

**Figure 2:**
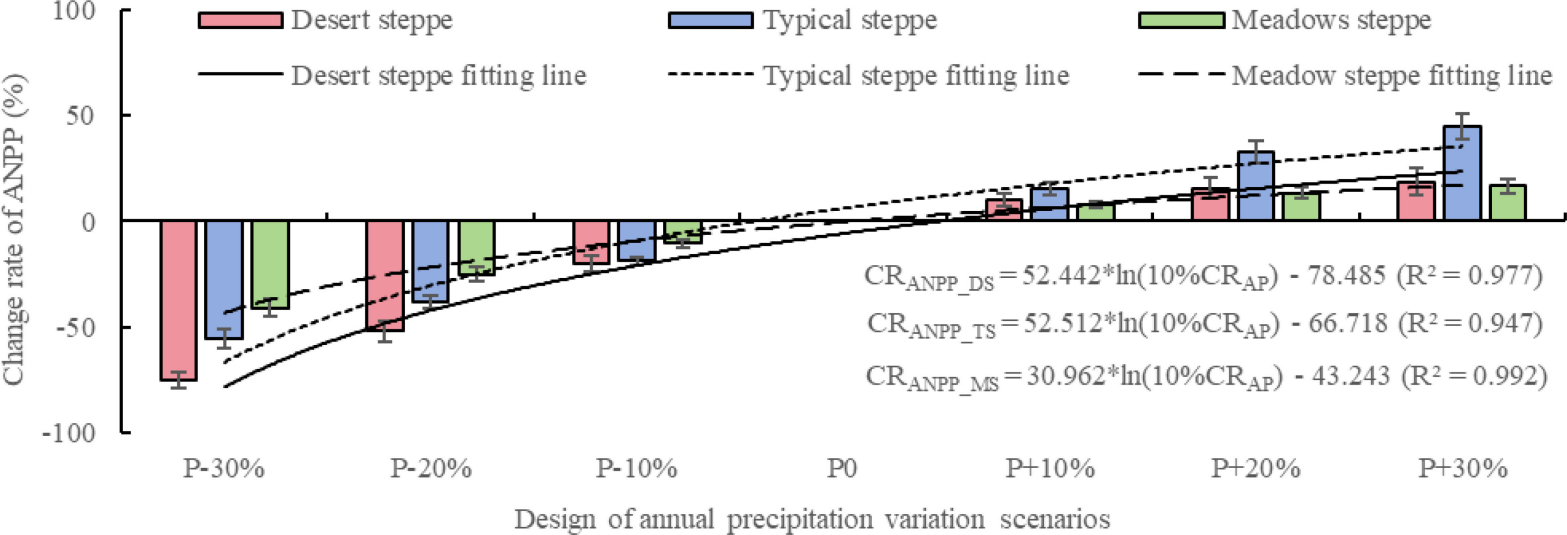
The impact of different annual precipitation changes on net primary productivity of three steppes based on APSIM model.

Based on the analysis of **Figure 2**, the three types of grassland vegetation exhibit heterogeneity in their response to annual precipitation. The response to a decrease in annual precipitation is significantly greater than the response to an increase. Under the scenario of decreased annual precipitation, all three types of steppes show a declining trend in ANPP, with desert steppe exhibiting the largest response in terms of ANPP reduction, followed by typical steppe and meadow steppe. Conversely, under the scenario of increased annual precipitation, all three types of steppes exhibit an increasing trend in ANPP, with typical steppe showing the strongest response, followed by desert steppe and meadow steppe. Specifically, the change rate of net primary productivity above ground for desert steppe (CR_ANPP_DS_), typical steppe (CR_ANPP_TS_), and meadow steppe (CR_ANPP_MS_) range from -74.97% to 18.79%, -55.75% to 44.66%, and -40.92% to 16.66%, respectively. When annual precipitation changes by -30%, -20%, -10%, 10%, 20%, and 30%, the CR_ANPP_DS_, CR_ANPP_TS_, and CR_ANPP_MS_ range from -74.97% to -40.92%, -25.08% to -51.94%, -10.60% to -19.91%, 7.81% to 15.73%, 13.39% to 32.97%, and 16.66% to 44.66%, respectively. Further analysis reveals that the CR_ANPP_DS_, CR_ANPP_TS_, and CR_ANPP_MS_ exhibit a positive logarithmic correlation with the rate of change in annual precipitation (CR_AP_) by 10%, with all R^2^ exceeding 0.94. In conclusion, CR_ANPP_MS_ is less affected by changes in annual precipitation.

**Figure 3:**
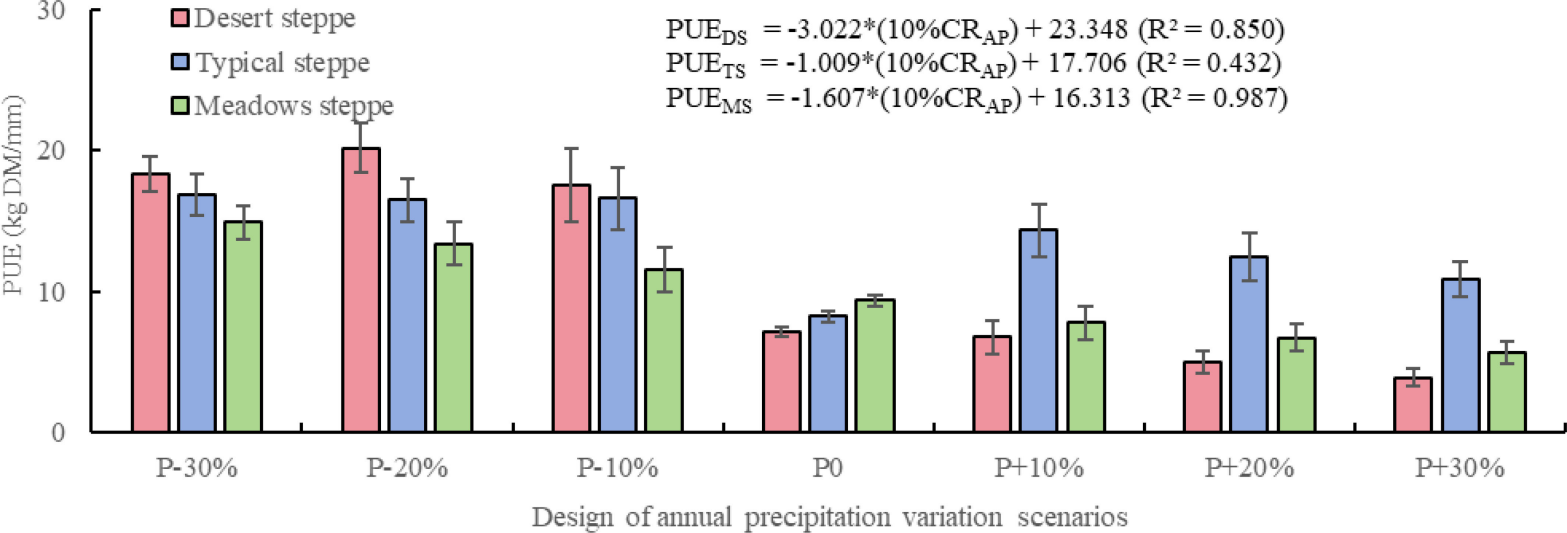
The impact of annual precipitation changes during development stages on PUE of three steppes based on APSIM model.

Based on the results of the precipitation utilization efficiency (PUE) shown in **Figure 3**, it can be observed that the impact of different annual precipitation on PUE is relatively small. When the annual precipitation decreases, the desert steppe has the highest PUE, followed by typical steppe, and meadow steppe has the lowest PUE. When the annual precipitation increases, the typical steppe has the highest PUE, followed by the meadow steppe, and the desert steppe has the lowest PUE. The PUE for desert steppe (PUE_DS_), typical steppe (PUE_TS_), and meadow steppe (PUE_MS_) range from 3.87 to 20.19, 8.20 to 16.82, and 5.61 to 14.88 kg DM/mm, respectively. When annual precipitation changes by -30%, -20%, -10%, 0, 10%, 20%, and 30%, the PUE_DS_, PUE_TS_, and PUE_MS_ range from 14.88 to 18.33, 13.39 to 20.19, 11.52 to 17.57, 7.13 to 9.35, 6.74 to 14.31, 5.00 to 12.41, and 3.87 to 10.87 kg DM/mm, respectively. Further analysis reveals that the PUE_DS_, PUE_TS_, and PUE_MS_ exhibit a positive linear decrease with the CR_AP_ by 10%.

### 3.2 Response of different types of grassland productivity to seasonal precipitation changes

**Figure 4:**
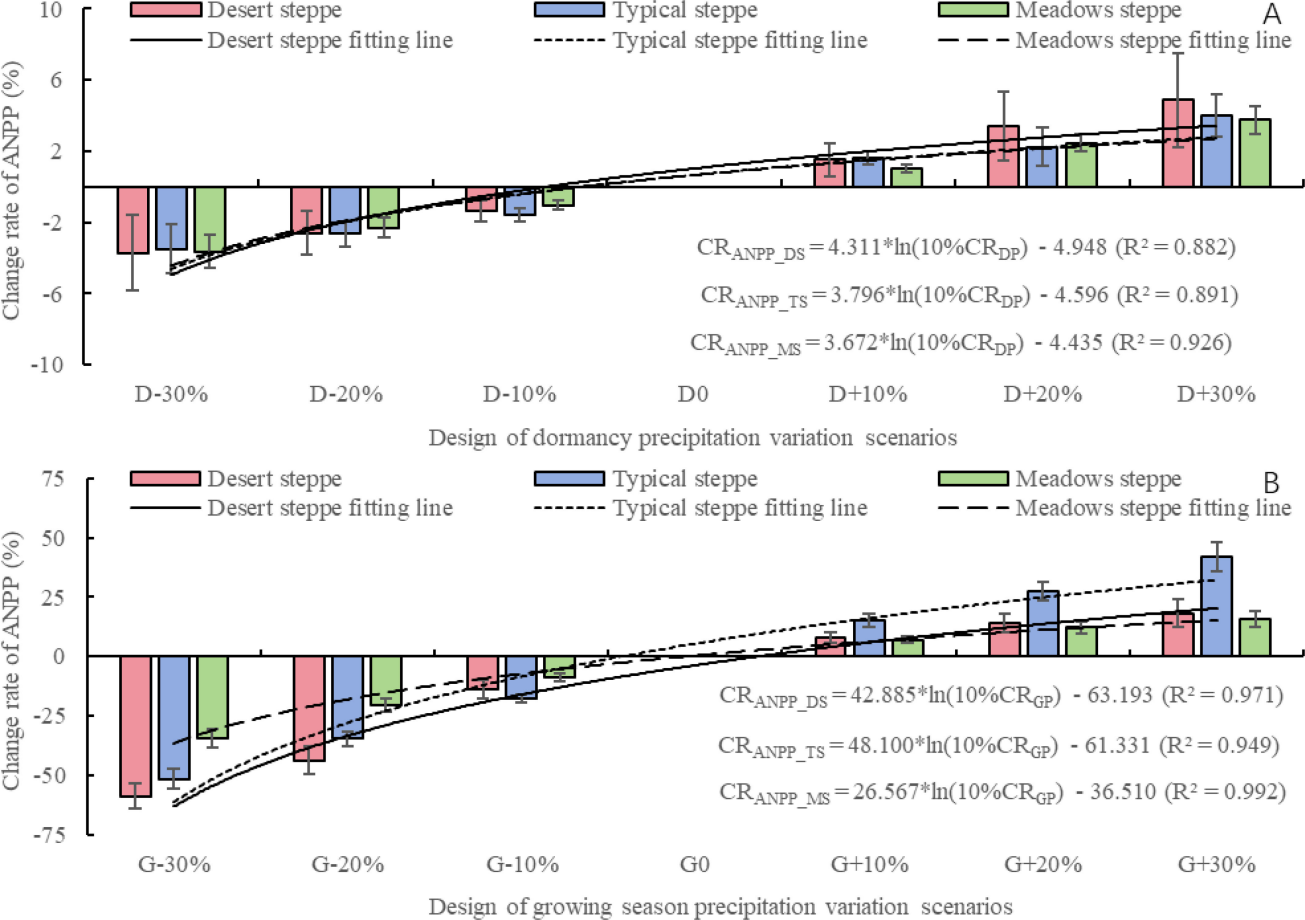
The impact of seasonal precipitation changes on aboveground net primary productivity of three steppes based on the APSIM model. **(A)** represents the dormancy precipitation scenario, and **(B)** represents the growing season precipitation scenario.

According to **Figure 4**, the three types of steppes show a consistent trend in responding to seasonal precipitation variations. It is observed that, under the same degree of change, the effect of precipitation changes during the growing season on ANPP is significantly greater than that during the dormancy. As shown in **Figure 4A**, when the dormancy precipitation changes by ±30%, the CR_ANPP_ from -3.71% to 4.87% for desert steppe, -3.49% to 4.03% for typical steppe, and -3.64% to 3.76% for meadow steppe. When the dormancy precipitation increases or decreases by the same degree, the contribution to ANPP is comparable. Specifically, when the dormancy precipitation changes by -30%, -20%, -10%, 10%, 20%, and 30%, the CR_ANPP_DS_, CR_ANPP_TS_, and CR_ANPP_MS_ are as follows: -3.49% to -3.71%, -2.31% to -2.64%, -1.03% to -1.57%, 1.03% to 1.63%, 2.23% to 3.39%, and 3.76% to 4.87%. Furthermore, as shown in **Figure 4B**, when the growing season precipitation changes by ±30%, the CR_ANPP_ from -58.89% to 18.14% for desert steppe, -51.57% to 41.89% for typical steppe, and -34.34% to 15.55% for meadow steppe. It is worth noting that, under the same degree of change, the impact of decreasing growing season precipitation on ANPP is greater than that of increasing growing season precipitation. Specifically, when the growing season precipitation changes by -30%, -20%, -10%, 10%, 20%, and 30%, the CR_ANPP_DS_, CR_ANPP_TS_, and CR_ANPP_MS_ are as follows: -58.89% to -34.34%, -43.82% to -20.56%, -17.68% to -8.96%, 7.04% to 15.19%, 12.19% to 27.55%, and 15.55% to 41.89%. Additionally, the magnitude of ANPP change is in the order of desert steppe > typical steppe > MS. Further analysis reveals that under the six precipitation scenarios, the CR_ANPP_DS_, CR_ANPP_TS_, and CR_ANPP_MS_ show a logarithmic positive correlation with a 10% change rate in dormancy precipitation (CR_DP_) and growing season precipitation (CR_GSP_). Among them, during the growing season precipitation, the CR_ANPP_ is highest for desert steppe, followed by typical steppe and meadow steppe. During the dormancy precipitation, the CR_ANPP_ is highest for desert steppe, followed by meadow steppe and then typical steppe. In conclusion, all three types of steppes show that changes in growing season precipitation have a greater impact on ANPP compared to dormancy precipitation. Additionally, decreasing precipitation has a greater impact on ANPP than increasing precipitation.

**Figure 5:**
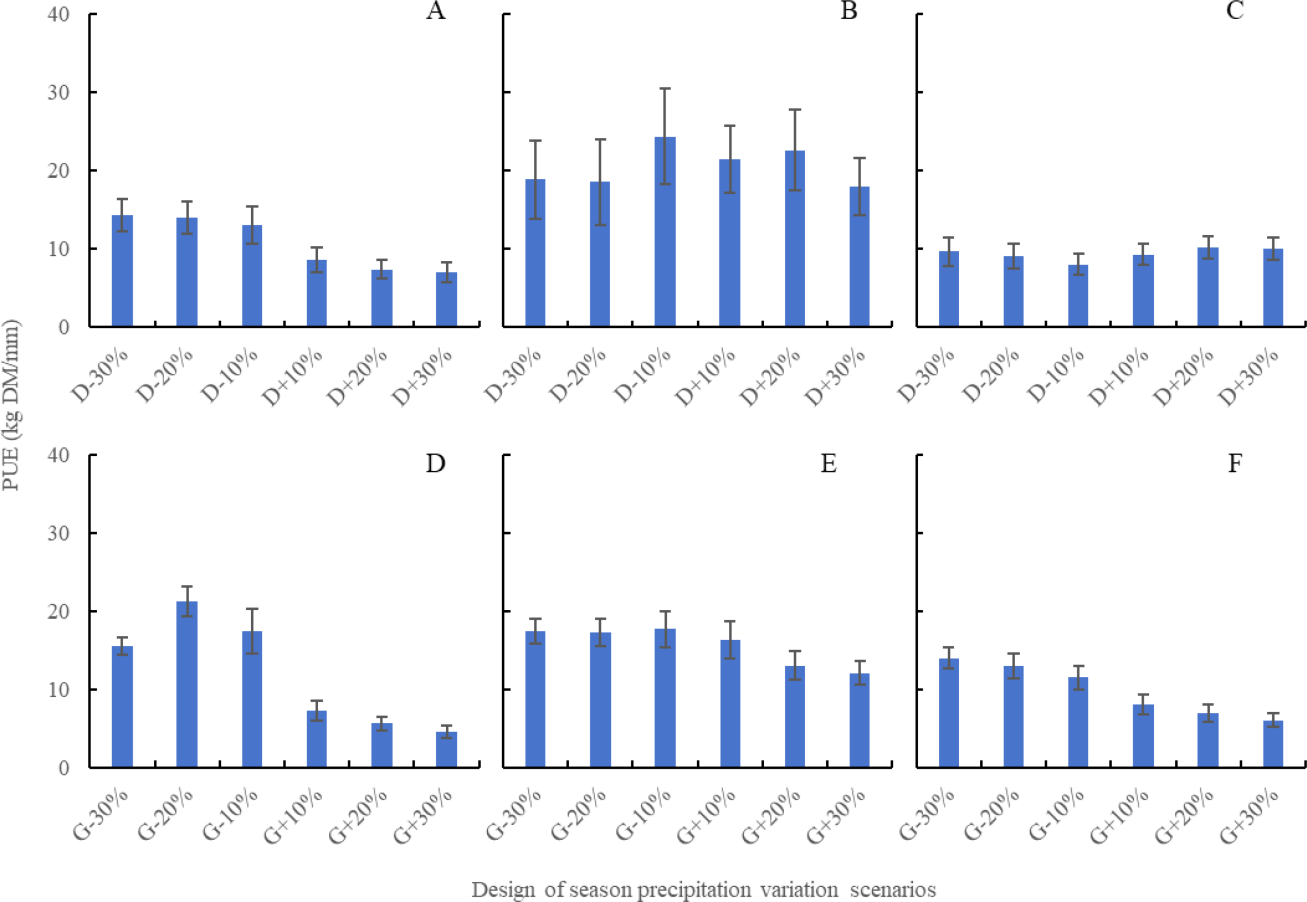
The impact of different seasonal precipitation changes on PUE of three steppes based on APSIM model. **(A)** represents the dormancy precipitation scenario design of desert steppe, **(B)** represents the dormancy precipitation scenario design of typical steppe, **(C)** represents the dormancy precipitation scenario design of meadows steppe, **(D)** represents the growing season precipitation scenario design of desert steppe, **(E)** represents the growing season precipitation scenario design of typical steppe, and **(F)** represents the growing season precipitation scenario design of meadows steppe.

The impact of precipitation variability during the growing season on ANPP is significantly greater than that during the dormancy, primarily due to higher precipitation levels in the former. This study further explores the effects of precipitation changes on ANPP using the PUE indicator. As illustrated in **Figure 5**, the discrepancies in contributions to ANPP from dormancy precipitation versus growing season precipitation diminish when assessed through PUE metrics. **Figures 5A–C** demonstrate that, among the different grassland types, typical steppe exhibited the highest PUE, followed by desert steppe, with meadow steppe showing the lowest PUE. Interestingly, desert steppe showed a higher PUE during dormancy precipitation compared to growing season precipitation, conversely to the patterns seen in typical steppe and meadow steppe. The PUE_DS_, PUE_TS_, and PUE_MS_ range between 6.97 to 14.34, 17.97 to 24.39, and 6.02 to 8.03 kg DM/mm, respectively. Variations in dormancy precipitation (-30%, -20%, -10%, 10%, 20%, and 30%) lead to changes in PUE rates among the three grassland types, which range from 9.67 to 18.87, 9.13 to 18.54, 8.03 to 24.39, 9.30 to 21.45, 7.39 to 22.60, and 6.97 to 17.97 kg DM/mm. **Figures 5D–F** reveal the influence of reduced growing season precipitation, where PUE follows the order: desert steppe > typical steppe > meadow steppe. Conversely, an increase in growing season precipitation results in the highest PUE for typical steppe. The PUE_DS_, PUE_TS_, and PUE_MS_ fall between 4.53 to 21.23, 12.07 to 17.70, and 6.02 to 13.97 kg DM/mm, respectively. PUE changes under different scenarios of growing season precipitation (-30%, -20%, -10%, 10%, 20%, and 30%) range from 13.97 to 17.44, 12.91 to 21.23, 11.44 to 17.70, 7.21 to 16.28, 5.57 to 13.02, and 4.53 to 12.07 kg DM/mm across the three grassland types. Additionally, when assessing the contributions of precipitation changes to ANPP based on PUE metrics, noteworthy differences emerge. For typical steppe and meadow steppe, the effect of dormancy precipitation changes stands out, with typical steppe demonstrating an equivalent contribution of dormancy precipitation to PUE as that of growing season precipitation.

### 3.3 Response of different types of grassland productivity to precipitation at different stages of the growing season

**Figure 6:**
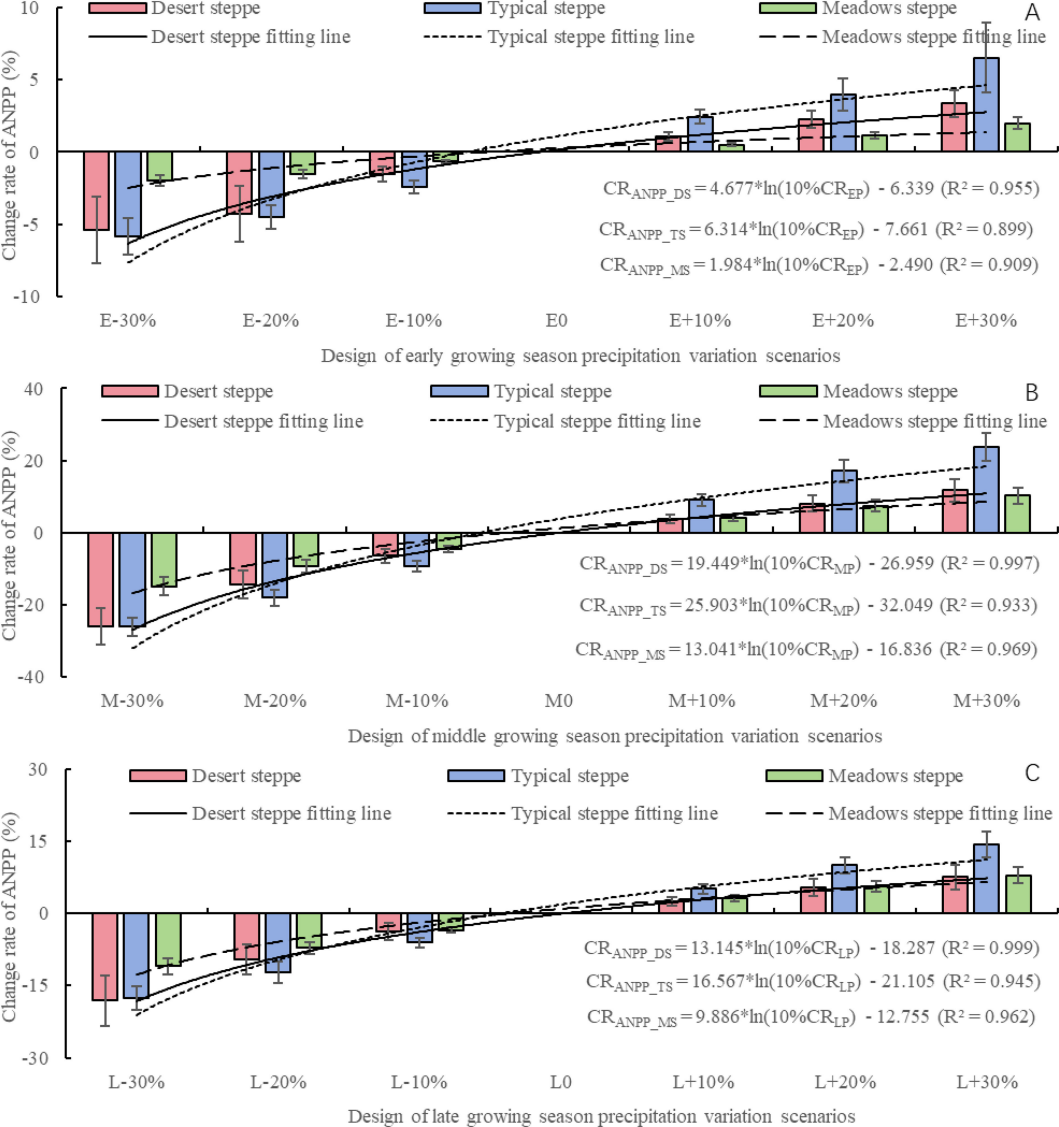
The impact of precipitation changes during different growth season development stages on aboveground net primary productivity of three steppes based on the APSIM model. **(A)** represents the early growing season precipitation scenario, **(B)** represents the middle growing season precipitation scenario, and **(C)** represents the late growing season precipitation scenario.

Based on the analysis of **Figures 4**, **5**, it is evident that increased precipitation during the growing season significantly enhances ANPP and PUE. Therefore, it is important to further investigate the impact of precipitation variations during the early, middle, and late stages of the growing season on ANPP and PUE. According to **Figure 6**, changes in precipitation during the early, middle, and late of the growing season precipitation all affect the ANPP of three types of steppes. Meadow steppe is least affected by precipitation variations during different stages of the growing season, with the CR_ANPP_ ranging from -5.38% to 3.36% during early growing season precipitation, -5.84% to 6.53% during middle growing season precipitation, and -1.95% to 1.98% during late growing season precipitation. The CR_ANPP_DS_, CR_ANPP_TS_, and CR_ANPP_MS_ during early growing season precipitation range from -5.38% to 3.36%, -5.84% to 6.53%, and -1.95% to 1.98%, respectively. During middle growing season precipitation, the CR_ANPP_DS_, CR_ANPP_TS_, and CR_ANPP_MS_ range from -25.98% to 11.84%, -26.04% to 23.67%, and -14.79% to 10.26% respectively. Similarly, during late growing season precipitation, the CR_ANPP_DS_, CR_ANPP_TS_, and CR_ANPP_MS_ range from -18.07% to 7.52%, -17.56% to 14.28%, and -10.99% to 7.90% respectively. For meadow steppe, the impact of precipitation variations during the growing season on meadow steppe ANPP is within ±15%, with the smallest impact during early growing season precipitation (<2%). During middle growing season precipitation and late growing season precipitation, reductions in precipitation contribute -9.49% and -7.23% to ANPP, while increases in precipitation contribute 7.26% and 5.56% to meadow steppe ANPP respectively. For typical steppe, the impact of precipitation variations during the growing season on ANPP is within ±30%, with the smallest impact during early growing season precipitation (<7%). During middle growing season precipitation and late growing season precipitation, reductions in precipitation contribute -17.78% and -11.94% to ANPP, while increases in precipitation contribute 16.61% and 9.78% to typical steppe ANPP respectively. For desert steppe, the impact of precipitation variations during the growing season on ANPP is also within ±30%, with the smallest impact during early growing season precipitation (<6%). During middle growing season precipitation and late growing season precipitation, reductions in precipitation contribute -15.58% and -10.45% to ANPP, while increases in precipitation contribute 7.94% and 5.14% to desert steppe ANPP respectively. By quantifying the relationship between precipitation variations during different stages of the growing season and ANPP for the three steppes, it is evident that CR_ANPP_DS_, CR_ANPP_TS_, and CR_ANPP_MS_ exhibits a logarithmic positive correlation with the rate of change in precipitation by 10% during any growing season stage. Regardless of the precipitation scenario during any stage of the growing season, the magnitude of the CR_ANPP_ follows the order of typical steppe, desert steppe, and meadow steppe. In summary, regardless of the precipitation scenario during any stage of the growing season, ANPP is most sensitive to precipitation changes during middle growing season precipitation, followed by late growing season precipitation, and has the least impact during early growing season precipitation.

**Figure 7:**
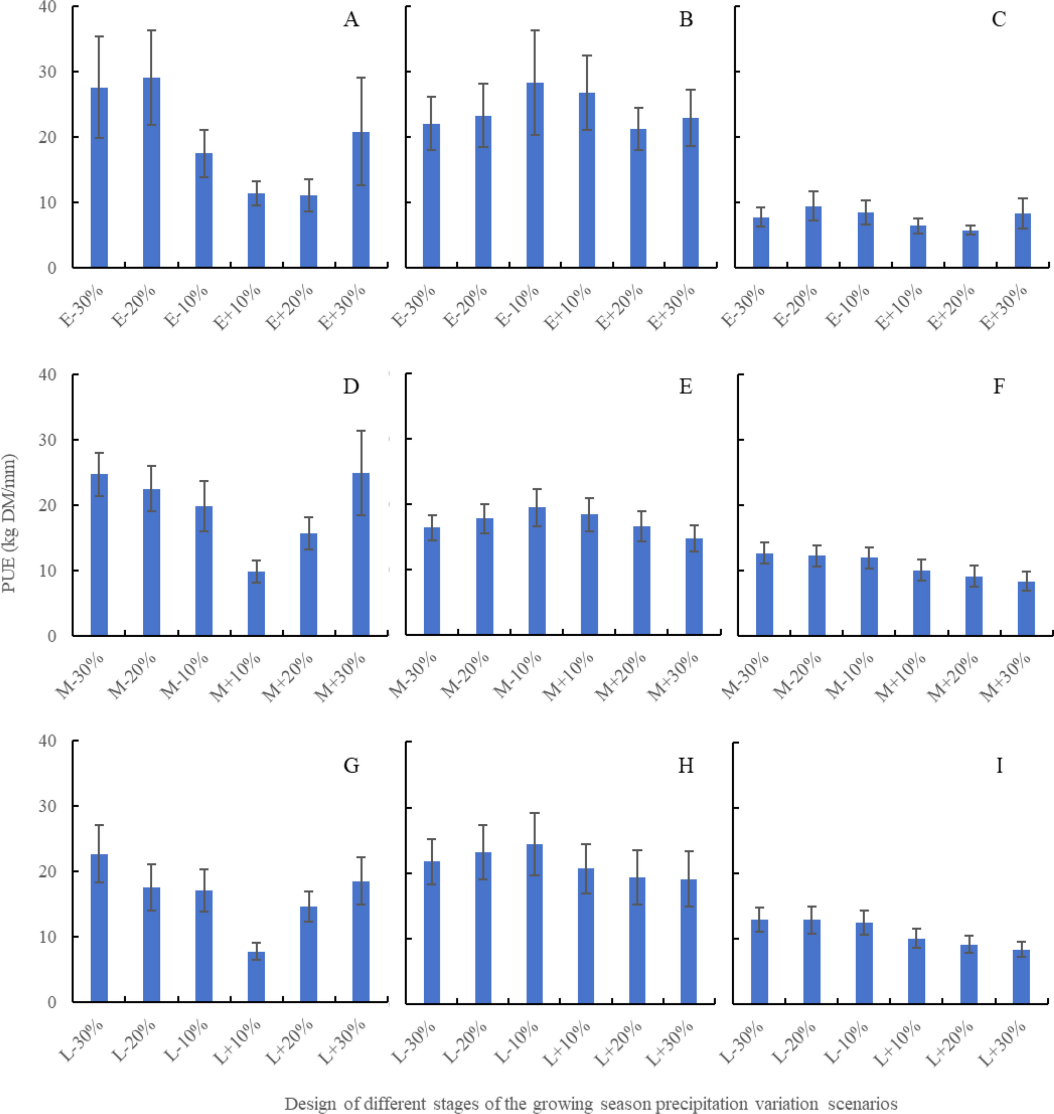
The impact of precipitation changes during different growth season development stages on PUE of three steppes based on APSIM model. **(A)** represents the early growing season precipitation scenario design of desert steppe, **(B)** represents the early growing season precipitation scenario design of typical steppe, **(C)** represents the early growing season precipitation scenario design of meadows steppe, **(D)** represents the middle growing season precipitation scenario design of desert steppe, **(E)** represents the middle growing season precipitation scenario design of typical steppe, **(F)** represents the middle growing season precipitation scenario design of meadows steppe, **(G)** represents the late growing season precipitation scenario design of desert steppe, **(H)** represents the late growing season precipitation scenario design of typical steppe, and **(I)** represents the late growing season precipitation scenario design of meadows steppe.

According to **Figure 7**, the influence of precipitation changes on the PUE of desert steppe varies between 11.13 to 29.06 kg DM/mm, 9.84~24.91 kg DM/mm, and 7.74~22.65 kg DM/mm in the early growing season precipitation, middle growing season precipitation, and late growing season precipitation, showing a general downward trend followed by an upward trend. The influence on the PUE of typical steppe ranges from 21.25 to 28.22 kg DM/mm, 14.72 to 19.46 kg DM/mm, and 19.10 to 24.38 kg DM/mm in the early growing season precipitation, middle growing season precipitation, and late growing season precipitation, respectively, showing an initial increase followed by a decrease. The influence on the PUE of meadow steppe decreases gradually, ranging from 5.76 to 9.43 kg DM/mm, 8.34 to 12.61 kg DM/mm, and 8.33 to 12.94 kg DM/mm in the early growing season precipitation, middle growing season precipitation, and late growing season precipitation, respectively. Under different precipitation scenarios in early growing season precipitation, the influence of decreased precipitation on PUE is greater than that of increased precipitation for all three types of steppes. Specifically, for desert steppe, the PUE ranges from 17.00 to 29.06 kg DM/mm (decreased precipitation) and 7.74 to 24.91 kg DM/mm (increased precipitation). For typical steppe, the PUE ranges from 16.43 to 28.22 kg DM/mm (decreased precipitation) and 14.72 to 26.71 kg DM/mm (increased precipitation). For meadow steppe, the PUE ranges from 7.71 to 12.94 kg DM/mm (decreased precipitation) and 5.76 to 10.04 kg DM/mm (increased precipitation). To summarize, for desert steppe, precipitation changes in the early growing season precipitation are most important. For typical steppe, precipitation changes throughout the entire growth season are important. For meadow steppe, precipitation changes in the middle growing season precipitation, and late growing season precipitation are most important.

## 4 Discussion

This study utilized the APSIM model as a research tool to determine key model parameter for three types of steppe communities. Validation results showed that the APSIM model can accurately simulate the dynamic changes in aboveground net primary productivity (ANPP) for these three types of grassland sites. Furthermore, this study analyzed the impact of different precipitation scenarios on grassland productivity.

The analysis of different steppe vegetation communities in response to annual precipitation variations indicated that vegetation productivity is influenced by the precipitation time scales and amount. Moreover, the response of vegetation productivity to annual precipitation varied among different types of steppe vegetation communities (Cong et al., 2013; Andrew et al., 2020; Liu et al., 2023). This study found that the response of vegetation productivity to a significant decrease in annual precipitation was greater than that to an increase in annual precipitation. This could be attributed to water deficiency being a major limiting factor for vegetation growth in the Inner Mongolia grassland ecosystem (Gao et al., 2013; Chen et al., 2014). Further decrease in precipitation could lead to cumulative drought effects, causing deepening of plant root systems, reduction in leaf size and quantity, and a significant decrease in ANPP (Andrew et al., 2020; Liu et al., 2022; Wu et al., 2022).

The study also analyzed the response of different steppe vegetation communities to seasonal precipitation variations. Previous research has shown that the coupling effect of temperature and precipitation, characterized by rising minimum temperatures during the growing season, can significantly increase ANPP in alpine meadows. Future “warm-wet” climate change may enhance grassland productivity in the agro-pastoral transitional area of Inner Mongolia (Mo et al., 2012). Building upon previous research, this study further analyzed the response of different steppe vegetation communities to seasonal precipitation variations. Although the influence of precipitation during the growing season on ANPP was greater than that during the dormancy, both periods were equally important in terms of precipitation utilization efficiency (PUE). This may be due to the supplemental soil water and groundwater resources provided by dormancy precipitation, which can reduce the risk of water stress during grass regrowth and promote vegetation growth (Chen et al., 2014; Liu et al., 2022). Our study indicated the response of steppe community to precipitation change is different (**Figures 2**-**4**). Firstly, the natural precipitation at the three sites were different, where the precipitation in meadow steppe (358.7mm) is highest while the lowest in typical steppe (281.5mm). Therefore, the typical steppe faced with the severest drought threat, while the growth of plants in meadow steppe was temperature constrained. Additional precipitation may contribute more to ANPP improvement in typical steppe. Compared with desert steppe, although the precipitation is ~40mm higher, the soil water holding capacity at desert steppe was the smallest with a high fraction of sand, indicating the poor water storage under the same condition (He and Wang, 2019), thus the contribution of increased precipitation may not as significant as that in typical steppe. Additionally, the community type at the three steppes also showed varied characteristics, where plants at desert steppe have smaller leaf thus lower radiation use efficiency, the PUE would be smaller compared with typical steppe. The combination effect of plant, precipitation and soil contributed to the varied response of the three steppes community to changed precipitation.

Additionally, the study analyzed the response of different steppe vegetation communities to seasonal precipitation variations. Previous research has indicated that autumn and spring phenology play an equally important role in regulating carbon balance (Liu et al., 2023). While climate warming delays autumn phenology, an increase in early growing season precipitation can advance the onset of autumn phenology. Moreover, the spatiotemporal variations in temperature and precipitation can lead to significant fluctuations in steppe community growth during the late growing season (Cong et al., 2013). This study refined the response of different steppe vegetation communities to precipitation during the early, middle, and late stages of the growing season. Typically, the growing season for typical and meadow steppes spans from March to October (Li et al., 2013), whereas for desert steppes, it ranges from May to October. In order to maintain consistency, this study set the growing season as April to September, which may introduce certain errors to the research findings. The results indicated that precipitation during the middle stage of the growing season contributed the most to ANPP in meadow and typical steppes, while precipitation during the early stage contributed the most to ANPP in desert steppes. Furthermore, a decrease in precipitation had the greatest impact on ANPP within the growing season for desert steppes, while its impact on ANPP within the growing season was relatively small for meadow and typical steppes. This could be attributed to the ample precipitation resources and small inter-annual variations in meadow steppes (Gao et al., 2013), as well as the dominance of deep-rooted grass species in these communities, which exhibit certain growth advantages under drought conditions (Chen et al., 2017; Liu M. et al., 2017).

Future research prospects: In terms of model optimization, the influence of factors such as model parameters, multi-parameter equivalence, and ecological environment on model accuracy should be considered (Zhao et al., 2014; He et al., 2017). In terms of application, it is necessary to quantify the impacts of different temperature, precipitation, and CO_2_ concentration coupling characteristics on grassland productivity (Qu et al., 2016; Liu WJ. et al., 2017; Guan et al., 2018). This should be done by combining with Geographic Information System (GIS), Remote Sensing (RS), and Global Positioning System (GPS) technology to achieve regional model simulation (Singh and Dhadse, 2021), formulate degraded grassland management decisions that adapt to climate change, evaluate carbon sink potential and grazing capacity, optimize grazing systems, and ensure sustainable utilization of grassland resources. In terms of scenario design, we need to note that the effect of extreme precipitation (for changes exceed +30% or fall below -30%) was not explored in our study. The drought under intensified decrease of precipitation may led to plant failure, while heavy precipitation may lead to increased runoff and leaching, which need to be studied in the future research.

## 5 Conclusion

1. Aboveground net primary productivity (ANPP) is more affected by precipitation during the growing season than during the dormancy. ANPP increases with increasing precipitation, and the rate of change in ANPP is positively correlated with the rate of change in annual precipitation (AP), seasonal precipitation (SP), and growing season precipitation (GSP) at a rate of 10%, 20%, and 30% respectively. Under the scenario of AP, precipitation show asymmetry, with a larger response from decreased precipitation than increased precipitation to productivity. The change rate of ANPP (CR_ANPP_) is the highest in typical steppe (TS) of AP and GSP, while the opposite is true in dormancy precipitation (DP). The CR_ANPP_ is the smallest in meadow steppe (MS) of AP and GSP, while it is relatively high in DP. The CR_ANPP_ is the relatively high in desert steppe (DS) of AP and GSP, while it is highest in DP.
2. When AP decreases, precipitation utilization efficiency (PUE) varies in the following order: DS, TS, and MS. Conversely, when AP increases, PUE varies in the following order: TS, MS, and DS. PUE shows the same importance for both DP and GSP. PUE in TS and MS increases with increasing DP, while the trend is opposite in DS. PUE increases with increasing GSP. PUE in MS remains unchanged with changes in early growing season precipitation (EGSP), while it decreases with increasing EGSP in DS and TS. PUE decreases with increasing middle growing season precipitation (MSSP) and late growing season precipitation (LGSP).

## Data Availability

The datasets presented in this article are not readily available because they need to be used in future work. Requests to access the datasets should be directed to the corresponding author.

## References

[B1] Andrew J. F. Ingrid J. S. Melinda D. S. Alan K. K. (2020). Precipitation amount and event size interact to reduce ecosystem functioning during dry years in a mesic grassland. J. Int. Manage. 26, 658–668. doi: 10.1111/gcb.14789 31386797

[B2] Bodner G. S. Robles M. D. (2017). Enduring a decade of drought, Patterns and drivers of vegetation change in a semi-arid grassland. J. arid environments 136, 1–14. doi: 10.1016/j.jaridenv.2016.09.002

[B3] Chen Q. Hooper D. U. Li H. Gong X. Y. Peng F. Wang H.. (2017). Effects of resource addition on recovery of production and plant functional composition in degraded semiarid grasslands. Oecologia 184, 13–24. doi: 10.1007/s00442-017-3834-3 28243743

[B4] Chen X. Q. Li J. Xu L. Liu L. Ding D. (2014). Modeling greenup date of dominant grass species in the Inner Mongolian Grassland using air temperature and precipitation data. Int. J. Biometeorology 58, 463–471. doi: 10.1007/s00484-013-0732-1 24065573

[B5] Cheng C. Dong C. Y. Guan X. L. Chen X. G. Wu L. Zhu Y. C.. (2024). CPSM, a dynamic simulation model for cucumber productivity in solar greenhouse based on the principle of effective accumulated temperature. Agronomy 14, 1242. doi: 10.3390/agronomy14061242

[B6] Cheng C. Li C. Li W. M. Ye C. Y. Wang Y. S. Zhao C. S.. (2023). Optimal path of the simulation model in horticultural crop development and harvest period. Trans. Chin. Soc. Agric. Eng. 39, 158–167. doi: 10.11975/j.issn.1002-6819.202303028

[B7] Cong N. Wang T. Nan H. J. Ma Y. C. Wang X. H. Ranga B. M.. (2013). Changes in satellite-derived spring vegetation green-up date and its linkage to climate in China from 1982 to 2010, a multimethod analysis. Global Change Biol. 19, 881–891. doi: 10.1111/gcb.2013.19.issue-3 23504844

[B8] Felton A. J. Knapp A. K. Smith M. D. (2018). Carbon exchange responses of a mesic grassland to an extreme gradient of precipitation. Oecologia 189, 565–576. doi: 10.1007/s00442-018-4284-2 30411149

[B9] Gao T. Yan W. Wu L. Chen Y. C. (2013). Variations of temperature and precipitation during growing-season of three major crops in Inner Mongolia under numerical simulation scenarios in the coming 30 years. Chin. J. Agrometeorology 34, 501–511. doi: 10.3969/i.issn.1000-6362.2013.05.001

[B10] Gao X. H. Liu Q. Wang J. (2022). Simulation of response of spring wheat yield to sowing date, nitrogen application and precipitation in dryland of Longzhong based on APSIM model. J. Triticeae Crops 42, 371–379. doi: 10.7606/i.issn.1009-1041.2022.03.14

[B11] Gong J. J. Liu Z. J. Zhu G. X. Shi D. Y. Zhang Z. T. Fu Z. Z.. (2023). Effects of climate change on maize productivity in China during 2015 to 2100 based on APSIM model. Trans. Chin. Soc. Agric. Eng. 39, 167–178. doi: doi:10.11975/j.issn.1002-6819.202207115

[B12] Guan K. Y. Good S. P. Caylor K. K. Medvigy D. Pan M. Wood E. F.. (2018). Simulated sensitivity of African terrestrial ecosystem photosynthesis to rainfall frequency, intensity, and rainy season length. Environ. Res. Lett. 13, 025013. doi: 10.1088/1748-9326/aa9f30

[B13] Guo Q. Hu Z. M. Li S. G. Yu G. R. Sun X. M. Zhang L. M.. (2015). Contrasting responses of gross primary productivity to precipitation events in a water-limited and a temperature-limited grassland ecosystem. Agric. For. Meteorology 214-215, 169–177. doi: 10.1016/j.agrformet.2015.08.251

[B14] Hao Y. B. Zhou C. T. Liu W. J. Li L. F. Kang X. M. Jiang L. L.. (2017). Aboveground net primary productivity and carbon balance remain stable under extreme precipitation events in a semiarid steppe ecosystem. Agric. For. Meteorology 240, 1–9. doi: 10.1016/j.agrformet.2017.03.006

[B15] He D. Wang E. (2019). On the relation between soil water holding capacity and dryland crop productivity. Geoderma 353, 11–24. doi: 10.1016/j.geoderma.2019.06.022

[B16] He D. Wang E. L. Wang J. Lilley L. Luo Z. K. Pan X. B.. (2017). Uncertainty in canola phenology modelling induced by cultivar parameterization and its impact on simulated yield. Agric. For. Meteorology 232, 163–175. doi: 10.1016/j.agrformet.2016.08.013

[B17] Holzworth D. P. Huth N. I. Devoil P. G. Zurcher E. J. Herrmann N. I. McLeanet G.. (2014). APSIM-Evolution towards a new generation of agricultural systems simulation. Environ. Model. Software 62, 327–350. doi: 10.1016/j.envsoft.2014.07.009

[B18] Hoover D. L. Rogers B. M. (2016). Not all droughts are created equal, the impacts of interannual drought pattern and magnitude on grassland carbon cycling. Global Change Biol. 22, 1809–1820. doi: 10.1111/gcb.2016.22.issue-5 26568424

[B19] Hossain M. L. Kabir M. H. Nila M. U. S. Rubaiyat A. (2021). Response of grassland net primary productivity to dry and wet climatic events in four grassland types in Inner Mongolia. Plant Environ. Interact. 2, 250–262. doi: 10.1002/pei3.10064 37284512 PMC10168099

[B20] Hou J. H. Li Q. Y. Yan P. Xu L. Li M. X. He N. P. (2023). Universal rule and regional variation of vegetation height assembly of typical grasslands in China. J. Plant Ecol. 16, rtac048. doi: 10.1093/jpe/rtac048

[B21] Iturrate-Garcia M. O'Brien M. J. Khitun O. Abiven S. Niklaus P. A. Schaepman-Strub G. (2016). Interactive effects between plant functional types and soil factors on tundra species diversity and community composition. Ecol. Evol. 6, 8126–8137. doi: 10.1002/ece3.2016.6.issue-22 27878083 PMC5108264

[B22] Krause A. Pugh T. A. M. Bayer A. D. Li W. Leung F. Bondeau A.. (2018). Large uncertainty in carbon uptake potential of land-based climate-change mitigation efforts. Global Change Biol. 24, 3025–3038. doi: 10.1111/gcb.2018.24.issue-7 29569788

[B23] Li X. Z. Han G. D. Guo C. Y. (2013). Impacts of climate change on dominant pasture growing season in Central Inner Mongolia. Acta Ecologica Sin. 33, 4146–4155. doi: 10.5846/stxb201207301082

[B24] Li Q. Y. Pan X. B. Zhang L. Z. Li C. Yang N. Han S.. (2018). Responses of aboveground biomass and soil organic carbon to projected future climate change in Inner Mongolian grasslands. Rangeland J. 40, 101–112. doi: 10.1071/RJ16074

[B25] Li P. Peng C. Wang M. Luo Y. P. Li M. X. Zhang K. R.. (2018). Dynamics of vegetation autumn phenology and its response to multiple environmental factors from 1982 to 2012 on Qinghai-Tibet Plat-eau in China. Sci. Total Environ. 637-638, 855–864. doi: 10.1016/j.scitotenv.2018.05.031 29763866

[B26] Liu M. Gong J. R. Pan Y. Luo Q. P. Zhai Z. W. Yang L. L.. (2017). Response of dominant grassland species in the temperate steppe of Inner Mongolia to different land uses at leaf and ecosystem levels. Photosynthetica 56, 921–931. doi: 10.1007/s11099-017-0711-6

[B27] Liu W. J. Li L. F. Biederman J. A. Hao Y. B. Zhang H. Kanget X. M.. (2017). Repackaging precipitation into fewer, larger storms reduces ecosystem exchanges of CO_2_ and H_2_O in a semiarid steppe. Agric. For. Meteorology 247, 356–364. doi: 10.1016/j.agrformet.2017.08.029

[B28] Liu M. Li Y. He B. Zhao W.W. (2023). Spatiotemporal dynamics of grassland phenology and sensitivity to extreme precipitation in autumn in Qinghai-Tibetan Plateau. Res. Soil Water Conserv. 30, 353–363+372. doi: 10.13869/i.cnki.rswc.2023.03.050

[B29] Liu X. X. Li Y. Wang J. Huang M. X. Bai M. Song Y.. (2022). Adaptability evaluation of staple crops under different precipitation year types in four ecological regions of inner Mongolia based on APSIM. Scientia Agricultura Sin. 55, 1917–1937. doi: 10.3864/j.issn.0578-1752.2022.10.004

[B30] Mo Z. H. Li Y. E. Gao Q. Z. (2012). Simulation on productivity of main grassland ecosystems responding to climate change. Chin. J. Agrometeorology 33, 545–554. doi: 10.3969/i.issn.1000-6362.2012.04.012

[B31] Petrie M. D. Peters D. P. C. Yao J. Blair J. M. Burruss N. D. Collins S. L.. (2018). Regional grassland productivity responses to precipitation during multiyear above- and below- average rainfall periods. Global Change Biol. 24, 1935–1951. doi: 10.1111/gcb.2018.24.issue-5 29265568

[B32] Piao S. Fang J. Zhou L. Tan K. Tao S. (2007). Changes in biomass carbon stocks in China’s grasslands between 1982 and 1999. Global Biogeochemical Cycles 21, 1–10. doi: 10.1029/2005GB002634

[B33] Probert M. E. Dimes J. P. Keating B. A. Dalal R. C. Strong W. M. (1998). APSIM's water and nitrogen modules and simulation of the dynamics of water and nitrogen in fallow systems. Agric. Syst. 56, 1–28. doi: 10.1016/S0308-521X(97)00028-0

[B34] Qie Y. D. Teng D. X. Lv G.H. (2019). Response of plant niche to soil moisture and salinity in an arid desert area of Xinjiang, China. Acta Ecologica Sin. 39, 2899–2910. doi: 10.5846/stxb201801180135

[B35] Qu X. M. Zhang X. B. Wang M. Zang C. X. Guan X. (2016). Effect of different CO_2_ concentrations on photosynthetic performance of alfalfa during branching stage. Acta Agrestia Sin. 24, 988–994. doi: 10.11733/i.issn.1007-0435.2016.05.009

[B36] Ru J. Y. Wan S. Q. Hui D. F. Song J. Wang J. (2022). Increased interannual precipitation variability enhances the carbon sink in a semi-arid grassland. Funct. Ecol. 36, 987–997. doi: 10.1111/1365-2435.14011

[B37] Saxton K. E. Rawls W. J. (2006). Soil water characteristic estimates by texture and organic matter for hydrologic solutions. Soil Sci. Soc. America J. 70, 1569–1578. doi: 10.2136/sssaj2005.0117

[B38] Shen B. B. Ding L. Ma L. C. Li Z. W. Pulatov A. Kulenbekov Z.. (2022). Modeling the leaf area index of Inner Mongolia grassland based on machine learning regression algorithms incorporating empirical knowledge. Remote Sens. 14, 4196. doi: 10.3390/rs14174196

[B39] Singh A. P. Dhadse K. (2021). Economic evaluation of crop production in the Ganges region under climate change, A sustainable policy framework. J. Cleaner Production 278, 123413. doi: 10.1016/j.jclepro.2020.123413

[B40] Susanne R. Müller C. Jens H. Isabelle W. Anne B. Benjamin L. B.. (2018). Modeling vegetation and carbon dynamics of managed grasslands at the global scale with LPJmL 3.6. Geoentific Model. Dev. 11, 429–451. doi: 10.5194/gmd-11-429-2018

[B41] Tian Y. Q. Chen Y. Ouyang S. N. Sun Y. (2020). The effect of carbon and nitrogen addition on soil organic matter mineralization in the semi-arid grassland of north China. Ecol. Environ. Sci. 29, 1101–1108. doi: 10.16258/j.cnki.1674-5906.2020.06.004

[B42] Wang B. Ding X. C. Huang J. B. Gong X. L. Zhu S. J. Wang G. Z.. (2017). Grid runoff parameters estimation and adjustment of GSAC model based on HWSD. Trans. Chin. Soc. Agric. Machinery 48, 250–256+249. doi: 10.6041/i.issn.1000-298.2017.09.031

[B43] Wang G. C. Huang Y. Wei Y. R. Zhang W Li T. T. Zhang Q. (2019). Climate warming does not always extend the plant growing season in Inner Mongolian grasslands, evidence from a 30-year in *situ* observations at eight experimental sites. J. Geophysical Research Biogeosciences 124, 2364–2378. doi: 10.1029/2019JG005137

[B44] Wang E. Robertson M. J. Hammer G. L. Carberry P.S. Holzworth D. Meinkeet H.. (2002). Development of a generic crop model template in the cropping system model APSIM. Eur. J. Agron. 18, 121–140. doi: 10.1016/S1161-0301(02)00100-4

[B45] Wang Z. P. Zhang X. Z. He Y. T. Shi P. L. Zu J. X. Niu B.. (2018). Effects of precipitation changes on the precipitation use efficiency and aboveground productivity of alpine steppe-meadow on northern Tibetan Plateau, China. Chin. J. Appl. Ecol. 29, 1822–1828. doi: 10.13287/j.1001-9332.201806.009 29974690

[B46] Wu L. Cheng C. Yang F. Y. Fan D. L. Sun X. W. (2024). Research on water and nitrogen optimization management of spring wheat under drought conditions in Bayannur, Inner Mongolia based on APSIM model. Chin. J. Agrometeorology 45, 461–471. doi: 10.3969/j.issn.1000-6362.2024.05.002

[B47] Wu L. Feng L. P. Zhang Y. Gao J. C. Wang J. (2017). Comparison of five wheat models simulating phenology under different sowing dates and varieties. Agron. J. 109, 1280–1293. doi: 10.2134/agronj2016.10.0619

[B48] Wu L. Liu H. Y. Liang B. Y. Zhu X. R. Cao J. Wang Q. M.. (2022). A process-based model reveals the restoration gap of degraded grasslands in Inner Mongolian steppe. Sci. Total Environ. 806, 151324. doi: 10.1016/j.scitotenv.2021.151324 34749967

[B49] Yuan Q. L. Yang J. (2021). Phenological changes of grassland vegetation and its response to climate change in Qinghai-Tibet plateau. Chin. J. Grassland 43, 32–43. doi: 10.16742/i.zgcdxb.20210029

[B50] Zhang L. Ren H. (2023). Estimation of grassland height using optical and SAR remote sensing data. Adv. Space Research Off. J. Committee Space Res. (COSPAR) 72, 4298–4310. doi: 10.1016/j.asr.2023.08.018

[B51] Zhang L. H. Xie Z. K. Zhao R. F. Zhang Y. B. (2018). Plant, microbial community and soil property responses to an experimental precipitation gradient in a desert grassland. Appl. Soil Ecol. 127, 87–95. doi: 10.1016/j.apsoil.2018.02.005

[B52] Zhao G. Bryan B. A. Song X. (2014). Sensitivity and uncertainty analysis of the APSIM-wheat model, Interactions between cultivar, environmental, and management parameters. Ecol. Model. 279, 1–11. doi: 10.1016/j.ecolmodel.2014.02.003

[B53] Zhu X. Y. Xu D. W. Xin X. P. Shen B. B. Ding L. Wang X.. (2020). The spatial-temporal distribution of different grassland types in Hulunber grassland based on remote sensing from 1992 to 2015. Scientia Agricultura Sin. 53, 2715–2727. doi: 10.3864/j.issn.0578-1752.2020.13.019

